# Evidence for the Crash Avoidance Effectiveness of Intelligent and Connected Vehicle Technologies

**DOI:** 10.3390/ijerph18179228

**Published:** 2021-09-01

**Authors:** Hong Tan, Fuquan Zhao, Han Hao, Zongwei Liu

**Affiliations:** 1 State Key Laboratory of Automotive Safety and Energy, Tsinghua University, Beijing 100084, China; th18@mails.tsinghua.edu.cn (H.T.); zhaofuquan@tsinghua.edu.cn (F.Z.); Hao@tsinghua.edu.cn (H.H.); 2 Tsinghua Automotive Strategy Research Institute, Tsinghua University, Beijing 100084, China

**Keywords:** road safety, technological efficacy, autonomous vehicle

## Abstract

The Intelligent and Connected Vehicle (ICV) is regarded as a high-tech solution to reducing road traffic crashes in many countries across the world. However, it is not clear how effective these technologies are in avoiding crashes. This study sets out to summarize the evidence for the crash avoidance effectiveness of technologies equipped on ICVs. In this study, three common methods for safety benefit evaluation were identified: Field operation test (FOT), safety impact methodology (SIM), and statistical analysis methodology (SAM). The advantages and disadvantages of the three methods are compared. In addition, evidence for the crash avoidance effectiveness of Advanced Driver Assistance Systems (ADAS) and Vehicle-to-Vehicle communication Systems (V2V) are presented in the paper. More specifically, target crash scenarios and the effectiveness of technologies including FCW/AEB, ACC, LDW/LDP, BSD, IMA, and LTA are different. Overall, based on evidence from the literature, technologies on ICVs could significantly reduce the number of crashes.

## 1. Introduction

According to the data from World Health Organization, 1.35 million people die each year due to crashes on the road. This large figure depicts the pessimistic situation regarding global road safety [1]. In 2018 and 2017, 6,735,000 and 6,453,000 traffic crashes occurred in the United States, which resulted in 33,919 and 34,560 deaths, respectively [2]. Meanwhile, there were 12,472,797 and 10,256,317 traffic crashes in China in 2019 and 2018, respectively, resulting in 62,763 and 63,194 deaths. Official crash data from China and the United States indicate that an improvement in road traffic safety is urgent [3]. Indeed, many governments have committed to reducing the number of traffic crashes as a development goal.

Electrification, intellectualization, connecting, and sharing are profoundly changing the future transportation methods of society. The Intelligent and Connected Vehicle (ICV) is regarded as an emerging high-tech solution for reducing road traffic crashes, a central concern of many governments. Governments around the world have introduced a large number of policies and regulations to promote the implementation of intelligent vehicles. In a recent policy issued by Shenzhen, China, after obtaining a registration certificate and driving license, an ICV can drive on the roads of a special economic zone [4]. This means that driverless vehicles can be legally operated in Shenzhen, China. In the “Automated Vehicles Comprehensive Plan” released by the US Department of Transportation, developing automated vehicles is part of the plan to improve the safety of the transportation system [5]. A few technologies found in ICVs have been incorporated into the European New Car Assessment Program (Euro-NCAP), US-NCAP, and China-NCAP. For example, Euro-NCAP has taken automatic emergency braking (AEB), Lane Departure Warning (LDW), and speed assist system (SAS) as objects for evaluation.

As mentioned, ICV technologies play an important role in the future of road traffic safety. In the long term, hundreds of millions of high-level ICVs will be driven on the road. However, the crash avoidance effectiveness of ICV technologies is not clear. It is crucial for the government and enterprises to understand the crash avoidance potential of different ICV technologies and to be aware of which ICV technologies can be developed and applied in the short term. Although a large number of researchers have carried out research on ICV technologies’ safety benefits and thus contributed to the field, differences remain between the studies. Based on a review of recent literature, this study was conducted to provide a comprehensive insight into the crash avoidance potential of various ICV technologies.

According to the literature, three research methods that are often employed by scholars to evaluate the crash avoidance effectiveness of ICV technologies are introduced in the next section. Then, the effectiveness of eight major ICV technologies is collected from hundreds of papers and reports. The eight ICV technologies focused on in this study are Forward Collision Warning (FCW), AEB, Adaptive Cruise Control (ACC), Lane Departure Warning (LDW), Lane Keeping Assist (LKA), Blind Spot Detection (BSD), Intersection Management Assist (IMA), and Left Turn Assist (LTA). This review will provide key support for the research on ICV technologies and the development of strategies for different countries in the future. Finally, conclusions are made to summarize the current situations and limitations of evaluation research on the safety impact of ICV technologies, and worthwhile research topics are proposed for the future. The results can enable governments and enterprises to make more comprehensive decisions on the development of ICV technologies.

## 2. Three Evaluation Method

**Figure 1:**
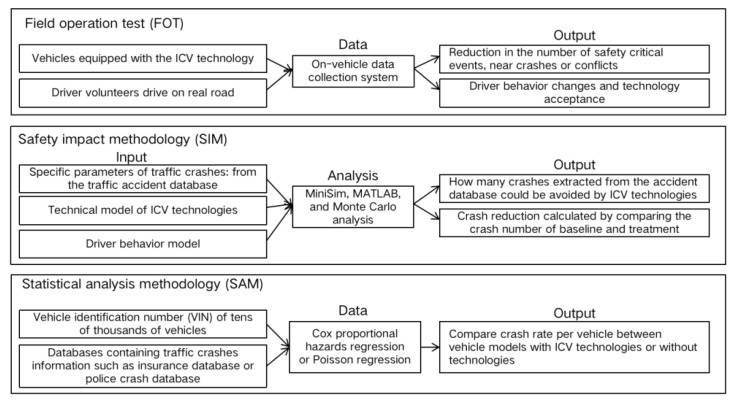
Three methods for evaluating the effectiveness of ICV technologies.

ICV technologies are in a stage of rapid development. The research on collision avoidance effectiveness of these ICV technologies has attracted the attention of many scholars. This study used “Google Scholar” and “Web of Science” to retrieve the relevant literature, and the keywords were related to autonomous vehicle technologies and safety impact. Literature related to the crash avoidance effectiveness of target technologies, mainly in 2010 and after, were the focus of this research. In addition, the relevant parameters and quantitative results in these papers were extracted and summarized. In general, three popular research methods were developed to evaluate the collision avoidance potential of ICV technologies. The three methods included Field operation test (FOT), safety impact methodology (SIM), and statistical analysis methodology (SAM), as shown in Figure 1. It is reasonable to use any of these three methods to study the safety benefit of ICV technologies, but there are obvious differences between them. The characteristics, advantages, and disadvantages of the three research methods are summarized and presented in the following text.

### 2.1. Field Operation Test (FOT)

A typical research process using a field operation test (FOT) involves recruiting drivers to drive vehicles equipped with specific ICV technologies on real roads, collecting relevant indicators through an on-vehicle data collection system, and comparing the safety performance data of a baseline group and a control fleet group in order to obtain the effectiveness of relevant ICV technologies. Technically, relevant technical samples need to be developed, which is the difficulty and challenge in the FOT study. The traffic flow environment in FOT is relatively uncomplicated because the technology may not apply to complex traffic scenarios. In terms of scale, the number of vehicles or drivers involved in FOT ranges from 20 to 200. To obtain more reliable results, the duration of FOT is usually very long, with some studies lasting for several months and the total driving mileage exceeding one million kilometers. From the perspective of safety performance data, traffic crashes generally do not occur during an FOT. The reduction in the number of safety-critical events, near-crashes, or conflicts in the baseline group and control group is used to indicate the improvement of safety brought about by the ICV technologies. In addition to the output of collision avoidance effectiveness of ICV technologies, it can also improve understanding of the driver’s use of technology and the driver’s behavioral changes, which is helpful for the future improvement of ICV technologies.

To study the collision avoidance effectiveness of BSD equipped on heavy trucks, in the FMCS-FOT experiment, 20 sample vehicles were developed by Schaudt et al. The driving mileage of the baseline fleet group and the control fleet group were 450,616 and 708,111 km, respectively. Finally, there are 33 BSD-related safety-critical events during the FMCS-FOT [6]. For studying the effectiveness of LDW and BSD equipped on light vehicles, Nodine et al. recruited 108 drivers to drive 16 vehicles equipped with LDW and BSD, with a total of 342,790 km. During the FOT, there were 1946 near-crashes [7]. The VOLVO IVI FOT was funded under the United States Department of Transportation (USDOT) Intelligent Vehicle Initiative (IVI). 100 Volvo trucks were organized into 3 fleets and equipped with advanced safety systems including ACC and AEB [8]. Over three years, more than 1000 drivers participated in the VOLVO IVI FOT, driving 16.3 million kilometers. The first European large-scale field operational test euroFOT focused on the safety impact of eight different ICV technologies. About 1000 vehicles drove a total of 34.86 million kilometers [9]. The results of the data analysis show positive effects on traffic safety. Meanwhile, euroFOT also pointed out the changes in driving behavior. Drivers mainly use ACC on motorways. The proportion of kilometers driven with active ACC reaches almost 50%.

However, there are some limitations with FOT research. First, the FOT evaluation can only be carried out after the development of prototype vehicles equipped with ICV technologies. Many more advanced ICV technologies cannot be studied through FOT because they have not yet been developed. Second, ICV technologies equipped on the prototype vehicle are often in the stage of laboratory research, which is different from the ICV technology put onto the market. The results may not represent the effectiveness of the ICV technology after its universal application. Third, the parameters of an ICV technology usually do not change in an FOT, so it is difficult to discuss the influence of different parameters of ICV technology on its crash avoidance effectiveness. Fourth, the FOT is usually carried out in a fixed road area. Thus, it is difficult to discuss the impact of different road conditions and weather conditions on technical effectiveness. Finally, the cost of FOT is relatively high. If the scale is too small, the credibility of the experiment is inadequate. On the contrary, if the scale is too large, the experiment will last too long, and the cost will be too high.

### 2.2. Safety Impact Methodology (SIM)

Generally, a complete SIM research project has three inputs, including the specific parameters of traffic crashes, the technical model of ICV technologies, and the driver behavior model. Then, software tools (such as MiniSim and MATLAB) and statistical techniques (such as Monte Carlo analysis) are also involved in SIM research. Usually, the specific parameters of the target crashes are extracted from a traffic crash database. The crash parameters include weather conditions, traffic flow, driving speed, braking parameters, and so on. Based on these parameters, simulation modeling and motion analysis can be carried out. The traffic crash databases often used for research are GES in the United States, GIDAS in Germany, FICA in Sweden and so on. Generally, the number of typical crashes used in a SIM study is between 500 and 1000. A technical model of and ICV technology is created by directly defining the main characteristics of the technology, such as sensor parameters and braking deceleration [10,11]. The other method uses the actual performance of the technology in the FOT to build a technical model [2,12]. To build the driver model, using a driving simulator to obtain data is the most common method. The research team usually recruit 20 to 100 drivers to use the driving simulator to collect vehicle driving data, driver reaction time, driver operational behavior, and other information in the experiment. There are two outputs of SIM research., One is how many crashes extracted from the crash database could be directly avoided by instituting ICV technologies. The other reports how much the safety parameters in the driving simulator under the specified technical scenario are improved compared with parameters without relevant ICV technologies, in order to indirectly calculate the crash avoidance effectiveness.

Sternlund et al. estimated the benefit to pedestrians if all vehicles in the United States were equipped with an automated braking system [13]. Crash characteristics were collected from three databases including PCDS, GES, and FARS, which are nationally representative databases collected by NHTSA. The AEB technology was modeled for two functions by detecting the pedestrian and applying emergency braking with a range of computational latencies (0–0.3 s), braking time-to-collision thresholds (0.5–1.5 s), and braking peak magnitudes (0.3–0.8 g). Guglielmi et al. estimated the safety benefits of heavy-vehicle crash warning applications relying on three sources [12]. National crash databases provide information about the driving conditions of the various target pre-crash scenarios. An Integrated Vehicle-Based Safety System field operational test generated data about driver/vehicle performance and system capability. The Monte Carlo technique was used to simulate the basic kinematics of driver/vehicle response to conflicts experienced during driving.

There are many advantages in using SIM to evaluate the safety benefits of ICV technologies. First, the effectiveness under different weather scenarios and different road scenarios can be studied by SIM. Gordon compared the effectiveness of LDW technology in urban and rural scenarios [14]. Second, the technical model of ICV technologies in SIM can be adjusted to meet the driver’s needs, which helps achieve a better parameter setting to maximize the crash avoidance ability. Riexenger et al. focused on the crashes that were not prevented by LDW/LDP in the hope of setting research priorities for next-generation road departure prevention systems [15]. Third, the impact of unexpected factors on the results can be excluded from the SIM study. The simulation times of SIM research can reach tens of thousands. Kusano conducted 24,882 simulations in a SIM study [16], while Gordon conducted 15,000 simulations [14]. There are so many simulations run that the researcher can throw out outliers and still have enough for a useful study. For the above reasons, SIM is widely used to evaluate the collision avoidance effectiveness of ICV technologies.

### 2.3. Statistical Analysis Methodology (SAM)

A typical research process using statistical analysis methodology (SAM) includes associating the vehicle identification number (VIN) of tens of thousands of vehicles with databases containing traffic crash information such as insurance databases or police crash databases. Then, Cox regression or Poisson regression is used to compare the crash rate per vehicle between vehicle models with and without ICV technologies. With the support provided by an automobile manufacturers, the vehicle identification number (VIN) can be decoded to provide the type of ICV technologies equipped on the vehicle. The insurance database and police crash database contain the details of the crash, such as speed limit, weather, road surface condition, and vehicle type.

In existing SAM research, it is common to use the VIN data from only one automobile manufacturer for research. Leslie analyzed the effectiveness of AEB, LDW, LKA, and other ICV technologies by using the VIN data on 3,785,419 vehicles of 22 GM models and the crash data reported by the police in 10 states of the United States [17]. BMW Automated Crash Notification system data (from January 2014 to November 2017) were merged with VIN data on 1,063,503 BMW passenger vehicles to identify vehicles that crashed [18]. In addition to matching VIN data with crash data, insurance data have also been used in some studies. Data on rear-end crashes in Sweden reported to insurance companies were used to calculate the effectiveness by comparing insurance data of the same Volvo vehicle model with and without the technology [19]. Cicchino used the VIN data from several automobile manufacturers [20]. VINs of General Motors, Mazda, Mercedes-Benz, and Volvo vehicles, equipped with optional crash avoidance technologies, including blind spot monitoring, were obtained from the manufacturers. Police-reported crash data were obtained from 26 states that released the VINs of the vehicles involved in crashes.

Compared with SIM and FOT, the advantage of SAM is that it enables an evaluation of the collision avoidance effectiveness of the large-scale use of ICV technologies in the real world. The results, based on a large number of practical data, can truly reflect the actual effectiveness of ICV technology applications. However, there are also shortcomings. As for the technology of interest, SAM can only evaluate the collision avoidance effectiveness of ICV technologies carried by the sold vehicle models of the automobile manufacturers. For more advanced ICV technology, it is impossible to evaluate with the SAM method because it has not yet been sold to consumers, similarly to FOT.

Generally speaking, SAM, FOT, and SIM are feasible methods to study the crash avoidance effectiveness of ICV technologies. In terms of variability, SIM can study the difference of crash avoidance effectiveness under different parameters of the same technology, therefore providing insight to improvethe technology or establish the parameters. SIM also has the ability to assess the crash avoidance potential of more advanced ICV technologies that have not yet been applied, because SIM can make assumptions about technical characteristics. From the output results, SAM can evaluate the actual crash avoidance benefits after the large-scale deployment of a technology. Supported by a large amount of data, the authenticity of SAM results is higher than that of SIM and FOT. However, the evaluation object of SAM is limited to the ICV technology equipped on the vehicle models that have been sold in the market. Thus, FOT is the best way to study any change in driver behavior. In the future, FOT could be used to obtain driver behavior data and technical performance characteristics as the input of SIM research.

## 3. Crash Avoidance Effectiveness

In the field of crash avoidance effectiveness evaluation, the research objects mainly focus on AEB, ACC, LDW, LKA, BSD, IMA, and LTA. Among these technologies, AEB, ACC, LDW, LKA, and BSD are kinds of advanced driver assistance systems. IMA and LTA are kinds of technologies based on Vehicle-to-Vehicle communication. Detailed research information including vehicle type, research method, sample size, target crash scenario, crash avoidance effectiveness, year, country, and author were collected from various related studies. Among these variables, vehicle type refers to passenger vehicle (PV) and heavy truck (HT). Research methods refer to the methods of SAM, FOT and SIM, which were discussed earlier. Sample size (SS) refers to the number of related crashes used in the study or the size of the FOT. The target crash scenario indicates the type of crash that can be avoided by the ICV technology. Crash avoidance effectiveness (Eff.) refers to the percentage of crash probability reduction in each vehicle if the technology is installed. In other words, it represents the percentage reduction in the number of crashes if all vehicles are equipped with the right technology. Most studies give a range of effectiveness, and the range is averaged to obtain the crash avoidance effectiveness of each study.

### 3.1. Advanced Driver Assistance Systems

In Germany, Australia, Switzerland, and the United States, scholars have conducted studies on the effectiveness evaluation of AEB and FCW. The target crash scenarios that AEB and FCW can avoid are rear-end crashes, cyclist crashes, and pedestrian crashes, as shown in Table 1. Most studies only focus on rear-end crashes. Some studies have paid an increased level of attention to pedestrian crashes and cyclist crashes. According to the available evidence, the effectiveness of AEB in avoiding target crash ranges from 18% to 72%. By using different research methods, differences emerge in the evaluation effectiveness. In the existing SAM studies, the effectiveness of AEB technology ranges from 27% to 46%. The output of SIM research is much higher, in the range of 40–72%. The result of SAM research is the actual benefit after large-scale application, which is related to the frequency of consumer use, driving style, and road conditions. However, the result of SIM research is the potential effectiveness of the application of AEB in an ideal situation, and some limiting factors are not fully considered. Compared with FCW, which only provides early warning in dangerous situations, AEB can provide active braking, so the crash avoidance effectiveness of AEB could be greatly improved. As one of the future development trends of V2V technology, FCW-V2V has also attracted scholars’ attention. Some studies have shown that the crash avoidance effectiveness of FCW is about 21%, while the effectiveness of FCW-V2V improves to 41%, benefitting from the improvement of vehicle perception distance brought about by V2V technology [12]. In addition, the effectiveness of AEB technology is also related to the driver’s feedback when AEB is working [21]. The study conducted by Geogri shows that if the driver did not give braking feedback to the system warning and system braking, the effectiveness only reached 64%; if the driver gave slight braking feedback, the effectiveness reached 72%. Finally, if the driver gave maximum braking feedback, the effectiveness would increase to 85%.

The target crash scenario that ACC technology can avoid is the rear-end crash. According to the available evidence in Table 2, the effectiveness of ACC in avoiding a rear-end crash ranges from 12% to 16%, which is a low level. The effectiveness of ACC with automatic emergency braking could be greatly improved to 45%, while the effectiveness of ACC with FCW could be slightly improved. The effectiveness of ACC technology is closely related to driver behavior and driver acceptance. Therefore, the FOT method is often used to study ACC technology.

Crashes that could be avoided by LKA and LDW methods are lane-departure-related crashes, including single crashes, front crashes, sideswipe same direction crashes, and sideswipe opposite direction crashes. The crash avoidance potential of LKA technology, which keeps the vehicle in the lane through active steering, is much higher than that of LDW technology, which only provides a warning when the vehicle deviates from the lane. According to the evidence in Table 3, the effectiveness of LDW is in the range of 10–48%. The effectiveness of LKA is in the range of 20–51%. In contrast, the crash avoidance effectiveness levels of the LDW, LKA with light steering, and LKA with aggressive steering are estimated to be 26%, 32%, and 37%, respectively [33].

The target crash scenario that could be avoided by BSD is the lane-change crash. According to the evidence in the Table 4, the crash avoidance effectiveness of BSD ranges between 14% and 58%. There are two evaluation studies on BSD-V2V, and the effectiveness values are 30% and 39%, respectively [12,37]. The main methods used to study the effectiveness of BSD are SAM and FOT. GM and BMW have introduced many BSD-equipped vehicles into the market, and a large amount of crash data and insurance data on BSD-equipped vehicles have been collected, allowing SAM research. At the same time, BSD technology is relatively mature, so it is not difficult to obtain an experimental vehicle equipped with BSD for FOT research.

### 3.2. Vehicle to Vehicle Communication Technologies

The V2V communication technology is considered to be an essential technology for intelligent vehicles in the future. Both the United States and China have taken V2V technology as the main development direction of intelligent vehicles in the future. IMA and LTA are the technologies of V2V communication technology that can directly avoid traffic crashes.

The target crash scenarios that could be avoided by IMA are intersection crashes, including straight crossing paths at non-signal (SCP), left turn into the path at non-signal (LTIP), right turn into the path at signal (RTIP), running a red light, and running a stop sign. In the evidence, the crash avoidance effectiveness of IMA is in the range of 23–67%, as shown in Table 5. There are two kinds of IMA technologies that have been widely studied by scholars; one only provides a warning in dangerous situations, the other is to provide active braking in dangerous situations. The crash avoidance effectiveness of IMA with warning ranges from 23% to 50%, while the effectiveness of IMA with directly braking ranges from 42% to 67%, which is more effective.

The target crash scenario that LTA technology can avoid is a left turn across path crash. Overall, the effectiveness of LTA is in the range of 32% to 60%, as shown in Table 6. Similar to IMA, there are two kinds of LTA technology, one of which only provides a warning, whilst the other can provide active braking in dangerous situations. There is a big difference between LTA with warning and LTA with active braking in terms of effectiveness. The crash avoidance effectiveness of LTA with warning is 32%, while the effectiveness of LTA with active braking ranges from 55% to 60%.

In fact, the actual effect of IMA and LTA on crash reduction is affected by many factors. First, the setting of key technical parameters such as the time to collision (TTC) threshold would affect the effectiveness of the technology. In the study conducted by Scanlon in 2016, the TTC thresholds of IMA were set to 2 s, 2.5 s, and 3 s, resulting in corresponding effectiveness of 19%, 32%, and 35%, respectively [41]. Second, the penetration rate of technology in the market will affect the effectiveness of crash avoidance. Only when enough vehicles are equipped with V2V communication equipment can IMA and LTA give full play to the collision avoidance ability. Third, driver behavior is also a key factor. The driver turning on the turn signal is the premise of LTA system activation. Only about 75% of drivers use the left turn signal when making a left turn [42]. The actual crash avoidance effect of LTA would be limited by the turning on rate, and the actual crash reduction percentage is only 75% of the potential.

It is worth noting that SIM is the main method to study the crash avoidance effectiveness of IMA and LTA. There are two reasons. On the one hand, compared with AEB, LDP, and other technologies, V2V communication technology is not yet mature enough, and there are no vehicle manufacturers currently equipping vehicles with V2V communication technology, therefore making it impossible to carry out SAM research. On the other hand, the cost of the SIM method is lower, and the technical parameters can be changed for comparative study.

## 4. Discussion

Methods used to study the crash avoidance effectiveness of ICV include FOT, SAM, and SIM. The three methods have varying advantages and disadvantages. Which method is selected to carry out the research depends on the ICV technology being studied and the resources that the researcher has available. If there are VIN data from the vehicle manufacturer and crash data from the police or insurance data from an insurance company, SAM is the most suitable research method. If a technical sample can be developed, FOT should be the first choice. At the same time, FOT can study the impact of ICV technologies on driver behavior and the driver’s acceptance of ICV technologies. In addition, SIM is an ideal method for studying the impact of future ICV technologies such as V2V on crash reduction. At the same time, in a SIM study, the technical parameters, scene parameters, and driver model parameters can be changed during the simulation, which helps to compare the technical effectiveness in different scenarios.

The effectiveness of crash avoidance in the real world is affected by many factors. Different parameter settings of the ICV technology would alter the effectiveness of the technology for crash avoidance. Generally speaking, AEB with active braking and LKA with active steering have higher crash avoidance effectiveness than FCW and LDW technologies, which only provide warnings. V2V communication technology will also empower BSD, LDW, and other technologies to improve the effectiveness of collision avoidance. In future, the large-scale application of connected vehicle technologies, such as V2V technology and vehicle to infrastructure communication technology, will make traffic safer. The number of crashes that the ICV technologies can reduce also depends on the market penetration rate, technology opening rate, driver behavior, and application scenarios. If the market penetration rate is not high enough or the ICV technology is not turned on by consumers, the technology will not be able to realize its potential. In fact, different countries have different numbers of crashes, and the proportions of crash types also differ. As a result, the number of crashes that each ICV technology can reduce varies across different countries. Therefore, each country should evaluate the benefits of ICV technology based on the country’s current traffic safety status in order to formulate a technology strategy from the national level.

## 5. Conclusions

We identified target crash types and effectiveness of eight ICV technologies through a systematic literature review. The target crash scenarios that can be avoided by different ICV technologies differ. Additionally, the crash avoidance effectiveness of different ICV technology is also differs as shown in Table 1, Table 2, Table 3, Table 4, Table 5 and Table 6. This database would provide key support for the studys on the safety impacts of these ICV technologies on road safety in countries around the world.

## Figures and Tables

**Table 1:** Evidence for the crash avoidance effectiveness of AEB and FCW.

Technology	Vehicle Type	Method	S.S	Crash Type	Eff.	Source
FCW	PV	SAM	4125	rear-end crash	21%	[17]
FCW	HT	SAM	3629	rear-end crash	21%	[22]
FCW-V2V	HT	SIM	40	rear-end crash	41%	[12]
AEB	PV	SAM	1,673,000	rear-end, single crash	66%	[23]
AEB	HT	SAM	84,000	rear-end crash	34%	[24]
AEB	HT	SIM	282	rear-end crash	40%	[25]
AEB	PV + HT	SIM	338	pedestrian	60%	[10]
EBA	PV	SIM	1103	rear-end crash	55%	[21]
AEB	PV	SIM	1103	rear-end crash	72%	[21]
AEB	PV	SIM	243	pedestrian	42%	[26]
AEB	PV	SIM	103	pedestrian, front, rear-end	18%	[11]
AEB	PV	SIM	1943	Cyclists and pedestrian	59%	[27]
AEB	PV	SAM	1178	rear-end crash	46%	[17]
AEB	PV	FOT	1021	rear-end crash	45%	[28]
AEB	PV	SAM	23,649	rear-end crash	43%	[20]
AEB	PV	SAM	454	rear-end crash	27%	[19]
AEB	PV	SAM	-	rear-end crash	38%	[29]

S.S means the sample size. Eff. means the crash avoidance effectiveness.

**Table 2:** Evidence for the crash avoidance effectiveness of ACC.

Technology	Vehicle Type	Method	S.S	Crash Type	Eff.	Source
ACC	PV	FOT	20	rear-end crash	13%	[30]
ACC	HT	FOT	100	rear-end crash	12%	[8]
ACC	HT	SIM	5000	rear-end crash	14%	[31]
ACC + FCW	PV	FOT	100	rear-end crash	16%	[9]
ACC + AEB	PV	SAM	35,401	rear-end crash	45%	[32]

**Table 3:** Evidence for the crash avoidance effectiveness of LDW and LKA.

Technology	Vehicle Type	Method	S.S	Crash Type	Eff.	Source
LDW	PV	SAM	22,65,000	Single, front, sideswipe	25%	[23]
LDW	HT	SAM	166,000	Sideswipe crash	10%	[24]
LDW	HT	SAM	5932	Sideswipe, head on, runoff road crash	48%	[34]
LDW	PV	FOT	108	Lane departure crash	19%	[7]
LDW	PV	SIM	478	Lane departure crash	26%	[33]
LDW	PV	SIM	76	Lane departure crash	47%	[14]
LDW	PV	SIM	128	Road departure crashes	27%	[15]
LDW	PV	SIM	478	Lane departure crash	17%	[35]
LDW	PV	SIM	478	Lane departure crash	29%	[16]
LDW	PV	SAM	5267	Lane departure crash	10%	[17]
LDW	PV	SAM	5433	Single-vehicle, head-on, and sideswipe crashes	18%	[36]
LKA	PV	SIM	478	Lane departure crash	35%	[33]
LKA	PV	SIM	128	Road departure crashes	51%	[15]
LKA	PV	SAM	2624	Lane departure crash	20%	[17]
LKA	PV	SAM	14,779	Lane departure crash	30%	[32]
LKA	PV	SAM	-	Head-on, single crashes	32%	[13]

**Table 4:** Evidence for the crash avoidance effectiveness of BSD.

Technology	Vehicle Type	Method	S.S	Crash Type	Eff.	Source
BSD	HT	FOT	33	lane change crash	58%	[6]
BSD	PV	FOT	108	lane change crash	41%	[7]
BSD	PV	SAM	15,507	lane change crash	14%	[18]
BSD	PV	SAM	4620	lane change crash	14%	[38]
BSD	PV	SAM	488	lane change crash	31%	[39]
BSD	PV	SAM	561	lane change crash	26%	[17]
BSD	PV	SAM	9716	lane change crash	32%	[32]
BSD-V2V	HT	SIM	28	lane change crash	39%	[37]
BSD-V2V	HT	SIM	140	lane change crash	30%	[12]

**Table 5:** Evidence for the crash avoidance effectiveness of IMA.

Technology	Vehicle Type	Method	S.S	Crash Type	Eff.	Source
IMA-Warning	PV	SIM	770	SCP crash	23%	[40]
IMA-Warning	PV	SIM	459	SCP crash	35%	[41]
IMA-Warning	PV	SIM	144	Intersection crash	48%	[42]
IMA-Warning	PV	SIM	96	Intersection crash	45%	[43]
IMA-Warning	PV	SIM	144	Intersection crash	50%	[44]
IMA-Braking	PV	SIM	792	SCP crash	67%	[45]
IMA-Braking	PV	SIM	770	SCP crash	42%	[40]
IMA-Braking	PV	SIM	459	SCP crash	49%	[41]
IMA-Braking	HT	SIM	84	SCP crash	64%	[12]
IMA-Braking	HT	SIM	40	Intersection crash	53%	[37]

**Table 6:** Evidence for the crash avoidance effectiveness of LTA.

Technology	Vehicle Type	Method	S.S	Crash Type	Eff.	Source
LTA-Warning	PV	SIM	501	Left turn across path crash	32%	[46]
LTA-Braking	PV	SIM	501	Left turn across path crash	60%	[46]
LTA-Braking	PV	SIM	96	Left turn across path crash	55%	[42]
LTA-Braking	PV	SIM	96	Left turn across path crash	56%	[2]

## Data Availability

All data generated or analyzed during this study are included in this published article and appendix. If more detailed data is needed, it’s available from the corresponding author.
